# An Analysis of the Correlation Between Component Placement and Functional Outcomes by Using Orthoscanogram in Primary Total Knee Arthroplasty

**DOI:** 10.7759/cureus.105203

**Published:** 2026-03-14

**Authors:** Harish DV, Raviraj Tantry, Amarnath Dasari, Aravind Devendrappa, Sravya Kotha, Mohammed Shahid

**Affiliations:** 1 Orthopaedics, Vydehi Institute of Medical Sciences and Research Centre, Bengaluru, IND; 2 Orthopaedics and Traumatology, Vydehi Institute of Medical Sciences and Research Centre, Bengaluru, IND; 3 Orthopaedic Surgery, Vydehi Institute of Medical Sciences and Research Centre, Bengaluru, IND

**Keywords:** component placement, functional outcome, knee society score, mechanical axis, orthoscanogram, total knee arthroplasty

## Abstract

Introduction

Total knee arthroplasty (TKA) is a well-established surgical treatment for advanced knee osteoarthritis, with the goals of relieving pain, restoring function, and improving the patient’s quality of life. Accurate alignment and optimal component placement are key determinants of implant longevity and postoperative functional outcomes following TKA. Restoration of the neutral mechanical axis of the lower limb is thought to minimize abnormal stresses on the prosthesis and surrounding bone. An orthoscanogram provides an accurate and reliable method for assessing the mechanical axis and overall axial alignment of the lower extremity. This study aimed to evaluate the correlation between component placement, assessed using orthoscanogram, and functional outcomes following primary TKA.

Methodology

This was a hospital-based comparative study conducted at Vydehi Institute of Medical Sciences and Research Centre, Bengaluru. A total of 60 patients with primary knee osteoarthritis who underwent primary TKA were included based on predefined inclusion and exclusion criteria. Full-length standing orthoscanograms were obtained preoperatively and postoperatively to assess mechanical axis alignment and component positioning using tibial zone classification. Functional outcomes were evaluated using the Knee Society Clinical Score (KSCS) and Knee Society Functional Score (KSFS) at a minimum follow-up of six months. Statistical analysis was performed to assess the association between mechanical axis alignment and functional outcomes.

Results

Postoperative orthoscanogram analysis demonstrated that patients in whom the mechanical axis passed through Zone 2 and the central (Zone C) of the tibial plateau showed significantly superior functional outcomes. According to the KSCS, the majority of patients achieved excellent to good results. Similarly, KSFS showed marked improvement postoperatively, particularly in patients with near-neutral mechanical alignment. A positive correlation was observed between optimal component placement, restoration of the mechanical axis, and improved functional outcome.

Conclusions

Accurate restoration of the mechanical axis and optimal component placement in primary TKA significantly influence postoperative functional outcomes. Orthoscanogram is a valuable and reliable tool for assessing lower limb alignment and component positioning. Postoperative mechanical axis passing through Zone 2 and the central zone is associated with excellent functional results. Emphasis on precise alignment during TKA can enhance patient satisfaction and potentially improve implant longevity.

## Introduction

Osteoarthritis of the knee is one of the most common degenerative joint disorders and a leading cause of pain, disability, and reduced quality of life, particularly among the elderly population. With increasing life expectancy, rising obesity rates, and greater functional demands of patients, the global burden of knee osteoarthritis continues to rise steadily [1]. When conservative management fails to provide adequate symptom relief, total knee arthroplasty (TKA) remains the most effective and definitive surgical treatment for end-stage knee arthritis [2]. TKA has evolved significantly since its introduction in the early 1970s, with substantial improvements in implant design, surgical techniques, fixation methods, and perioperative care. The primary goals of TKA are pain relief, restoration of knee function, correction of deformity, and long-term implant survival [3]. Despite these advancements, a proportion of patients continue to report suboptimal functional outcomes or dissatisfaction following TKA, underscoring the need to identify factors that influence the success of TKA [4].

Proper alignment and accurate component placement are widely regarded as critical determinants of successful outcomes following TKA. Restoration of the neutral mechanical axis of the lower limb is traditionally considered essential for even load distribution across the prosthesis and surrounding bone [5]. The mechanical axis of the lower limb is defined as a straight line drawn from the center of the femoral head to the center of the ankle joint, normally passing through the center of the knee joint. Deviation from this alignment can result in abnormal stress distribution, increased polyethylene wear, component loosening, instability, and early implant failure [6,7]. Several studies have demonstrated that malalignment exceeding ±3 degrees from the neutral mechanical axis is associated with increased rates of implant wear and reduced survivorship [8].

Varus or valgus malalignment leads to asymmetric loading of the tibial component, resulting in progressive bone loss and mechanical failure over time [9]. Consequently, achieving optimal axial alignment and precise positioning of the femoral and tibial components remains a key objective during TKA. Accurate assessment of lower limb alignment is essential both preoperatively and postoperatively. Conventional knee radiographs, while useful, are limited in their ability to assess global limb alignment [10] accurately. Full-length standing radiographs, commonly referred to as orthoscanograms or scannograms, provide a comprehensive evaluation of the mechanical axis by visualizing the hip, knee, and ankle joints in a single image [11]. Orthoscanogram enables precise measurement of coronal plane alignment and identification of the zone through which the mechanical axis passes across the tibial plateau, thereby enabling objective assessment of alignment restoration following TKA [12].

The Knee Society Scoring System is a widely accepted and validated tool for evaluating outcomes following TKA. It separately assesses knee clinical parameters such as pain, stability, and range of motion, as well as functional aspects including walking distance and stair climbing, allowing a comprehensive evaluation of surgical success [13]. Correlating radiological alignment parameters measured on orthoscanogram with functional outcome scores provides valuable insights into the clinical significance of component positioning. Although several studies have evaluated alignment techniques and navigation systems in TKA, there remains limited data correlating postoperative mechanical axis alignment, as assessed by orthoscanogram, with functional outcomes in the Indian population. Therefore, the present study was undertaken to evaluate the correlation between component placement and functional outcome using orthoscanogram in patients undergoing primary TKA, to reinforce the importance of accurate alignment in achieving optimal postoperative results.

## Materials and methods

Study design and setting

This prospective observational cohort study evaluated the correlation between postoperative mechanical axis alignment and functional outcomes following primary TKA. A prospective observational design was chosen to allow standardized patient selection, objective radiological assessment using orthoscanogram, and systematic postoperative functional evaluation using validated scoring systems [14]. The study was conducted in the Department of Orthopaedics at Vydehi Institute of Medical Sciences and Research Centre, Bengaluru, a tertiary-care referral hospital with dedicated arthroplasty services. The study period extended from July 2022 to August 2024, including patient recruitment, surgical intervention, postoperative rehabilitation, and follow-up evaluation. The study population comprised patients diagnosed with advanced primary knee osteoarthritis requiring TKA who presented to the orthopedic outpatient department during the study period. Eligible patients were consecutively recruited after fulfilling the inclusion and exclusion criteria and after providing written informed consent.

Sample size calculation

The sample size was calculated based on the primary outcome variable: the correlation between postoperative mechanical axis alignment and the Knee Society Functional Score (KSFS) [8,15]. The sample size was determined using Fisher’s Z transformation-based sample size calculation for correlation studies, based on previous literature evaluating alignment and functional outcomes following TKA. Using this calculation, the minimum required sample size was determined to be 52 patients. To account for possible loss to follow-up and incomplete data, the sample size was increased to 60 patients, all of whom were included in the final analysis.

Eligibility criteria

Inclusion Criteria

Patients aged 45 years or older diagnosed with primary osteoarthritis of the knee (Kellgren-Lawrence grade III or IV) and undergoing primary unilateral cemented TKA were included in the study. Only patients who were able to stand for full-length orthoscanogram imaging and were willing to comply with the postoperative rehabilitation protocol and follow-up schedule were recruited [2,3].

Exclusion Criteria

Patients were excluded if they had secondary osteoarthritis due to inflammatory arthritis (such as rheumatoid arthritis), post-traumatic arthritis, or metabolic bone disease, a history of previous major surgery on the affected knee or ipsilateral lower limb, or severe extra-articular deformity requiring complex reconstruction. Additional exclusion criteria included revision knee arthroplasty, neurological disorders affecting gait or lower limb function, active infection around the knee joint or systemic infection, BMI greater than 35 kg/m², severe medical comorbidities contraindicating major surgery, and inability to complete a minimum six-month follow-up [4,8].

Preoperative evaluation

All patients underwent a detailed preoperative clinical evaluation that included a comprehensive history, physical examination, assessment of deformity, range of motion, ligament stability, and gait pattern. Radiological evaluation consisted of weight-bearing anteroposterior and lateral radiographs of the knee, and the severity of osteoarthritis was graded using the Kellgren-Lawrence classification [16]. Additionally, a full-length standing orthoscanogram of both lower limbs was obtained to evaluate coronal plane alignment and determine the mechanical axis of the lower extremity.

Orthoscanograms were obtained with patients standing erect and bearing equal weight on both lower limbs, with the patellae facing forward to minimize rotational error. The mechanical axis of the lower limb, also known as Maquet’s line, was defined as a straight line drawn from the center of the femoral head to the center of the ankle joint [5]. The tibial plateau was divided into seven zones (0, 1, 2, C, 3, 4, and 5), and the zone through which the mechanical axis passed was recorded both preoperatively and postoperatively [10,11]. Orthoscanogram is widely regarded as the gold standard method for evaluating coronal alignment of the lower limb following total knee arthroplasty [17].

Surgical technique

All patients underwent cemented primary TKA under spinal or general anesthesia using a standard medial parapatellar approach. Surgeries were performed by experienced orthopedic surgeons using conventional instrumentation. Posterior cruciate ligament sacrifice was performed in all cases, and posterior-stabilized or ultra-congruent implants were used according to institutional protocol. Bone resections were made using standard anatomical landmarks with the aim of restoring neutral mechanical alignment. Intraoperative soft-tissue balancing was performed when necessary to achieve stable and symmetrical flexion and extension gaps. A pneumatic tourniquet was used in all cases, and particular attention was given to accurate component positioning to achieve postoperative mechanical axis alignment as close to neutral as possible [6,9].

Postoperative management and rehabilitation

All patients followed a standardized postoperative rehabilitation protocol. Early mobilization was initiated on the first postoperative day, including quadriceps strengthening exercises and progressive range-of-motion exercises. Weight bearing was gradually advanced as tolerated. Routine thromboprophylaxis and multimodal pain management strategies were administered. Sutures were removed on the 10th postoperative day, after which patients were discharged with instructions regarding home-based physiotherapy and scheduled follow-up visits.

Patients were reviewed at regular intervals, and both clinical and radiological evaluations were performed at six months postoperatively. A standing orthoscanogram was obtained during follow-up to assess the final mechanical axis alignment and component positioning. The postoperative mechanical axis zone was documented and compared with the preoperative alignment. Functional outcomes were assessed using the Knee Society Scoring System, which consists of two components: the Knee Society Clinical Score (KSCS), evaluating pain, stability, and range of motion, and the KSFS, assessing walking distance and stair-climbing ability. The Knee Society Scoring System is a validated and widely used outcome measure in studies evaluating TKA [13].

Statistical analysis

All data were entered into Microsoft Excel and analyzed using SPSS Statistics version 22.0 (IBM Corp., Armonk, NY). Quantitative variables were expressed as mean ± standard deviation (SD), while categorical variables were expressed as frequencies and percentages. Student’s t-test and chi-square test were used to analyze continuous and categorical variables, respectively. Correlation analysis was performed to evaluate the relationship between the mechanical axis zone and functional outcome scores. A p-value < 0.05 was considered statistically significant. Statistical methods were selected according to established principles of orthopedic outcome research methodology [18].

Ethical considerations

Ethical approval for the study was obtained from the Institutional Ethics Committee before the commencement of patient recruitment. Written informed consent was obtained from all participants. The confidentiality of patient data was strictly maintained throughout the study in accordance with ethical research guidelines and the principles of the Declaration of Helsinki.

## Results

**Figure 1:**
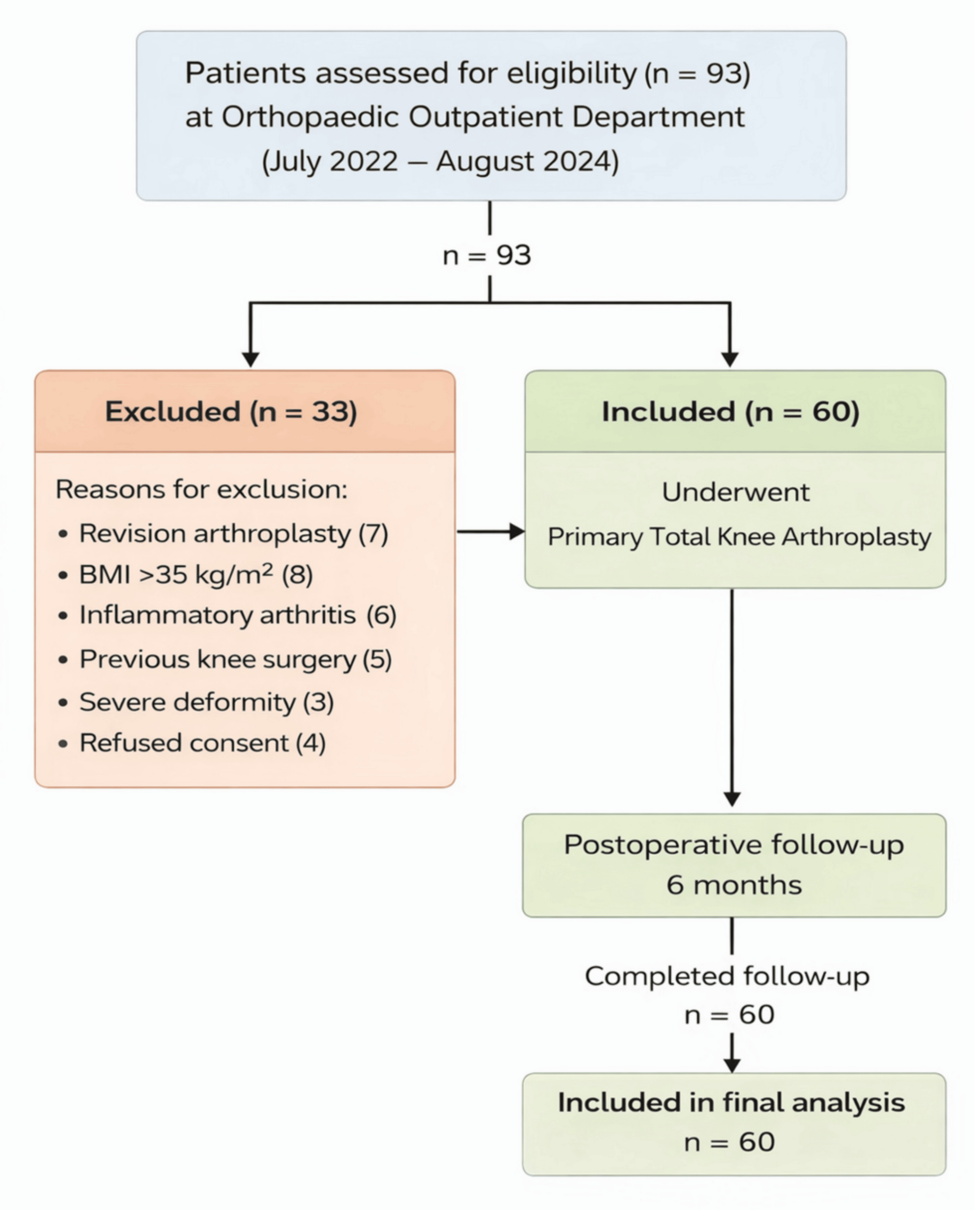
Flow diagram depicting patient selection

A total of 60 patients who underwent primary TKA were included in the study and were available for final evaluation at a minimum follow-up of six months (Figure 1). All patients completed the clinical, functional, and radiological assessments. The study population comprised patients aged between 45 and 75 years, with the majority belonging to the 61-70 years age group. There was a slight female predominance, consistent with the higher prevalence of knee osteoarthritis among elderly women. Most patients presented with varus deformity, which is the most common alignment abnormality associated with primary knee osteoarthritis (Table 1) [1,7]. A similar demographic distribution has been reported in previous large series of primary TKA [4].

**Table 1:** Demographic and preoperative clinical characteristics (n = 60)

Parameter	Category	Number of patients	Percentage (%)
Age group, years	45–50	6	10
	51–60	18	30
	61–70	28	46.7
	>70	8	13.3
Sex	Male	26	43.3
	Female	34	56.7
Side operated	Right	32	53.3
	Left	28	46.7
Preoperative deformity	Varus	49	81.7
	Valgus	11	18.3

**Figure 2:**
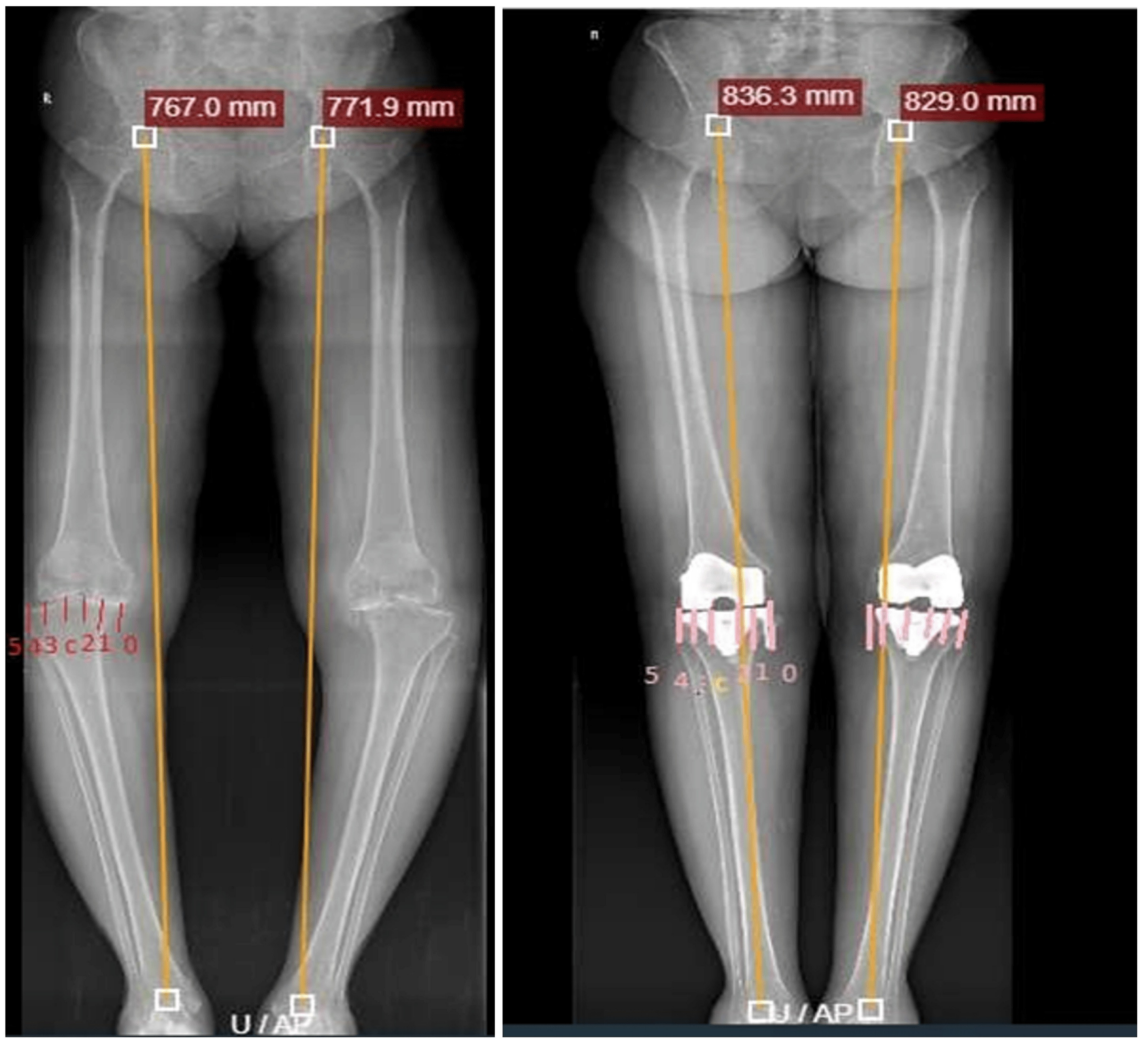
A case showing the distribution of mechanical axis zones pre- and postoperatively

Preoperative orthoscanograms revealed that the mechanical axis passed predominantly through zones 0, 1, and 3, indicating significant malalignment in most patients. Postoperative orthoscanograms demonstrated significant correction of the mechanical axis, with a majority passing through Zone 2 and Zone C, reflecting near-neutral alignment (Table 2, Figure 2). Postoperative correction toward Zone 2 and Zone C has been shown to represent optimal coronal alignment following TKA [6,8].

**Table 2:** Distribution of mechanical axis zones pre- and postoperatively

Mechanical axis zone	Preoperative (n)	Postoperative (n)
Zone 0	14	2
Zone 1	18	5
Zone 2	9	22
Zone C	6	21
Zone 3	10	7
Zone 4/5	3	3

There was a statistically significant improvement in both KSCS and KSFS at the six-month follow-up compared to preoperative values (Table 3). These findings are consistent with previous outcome studies reporting marked improvement in Knee Society Scores following successful TKA [3].

**Table 3:** Comparison of Knee Society Scores pre- and postoperatively SD: standard deviation

Score	Preoperative (mean ± SD)	Six-month postoperative (mean ± SD)	P-value
Knee Society Clinical Score	41.6 ± 6.4	89.3 ± 7.2	<0.001
Knee Society Functional Score	39.2 ± 7.1	86.7 ± 8.0	<0.001

Patients were grouped based on postoperative mechanical axis alignment. Those in whom the mechanical axis passed through Zone 2 and Zone C demonstrated superior functional outcomes compared to patients with residual malalignment. More than 85% of patients with the axis passing through Zone 2 and Zone C achieved excellent functional outcomes, whereas patients with residual varus or valgus alignment showed comparatively lower scores. This correlation was statistically significant (p < 0.05) (Table 4).

**Table 4:** Functional outcomes based on postoperative mechanical axis zone

Mechanical axis zone	Excellent	Good	Fair	Poor
Zone 2	20	2	0	0
Zone C	19	2	0	0
Other zones	8	7	1	1

The high proportion of excellent outcomes supports the importance of achieving optimal mechanical alignment during TKA (Table 5) [9,10]. No major complications such as infection, implant loosening, periprosthetic fracture, or revision surgery were observed during the follow-up period. Minor postoperative pain and transient stiffness were noted in a few patients and resolved with physiotherapy.

**Table 5:** Overall functional outcomes at the six-month follow-up

Outcome category	Number of patients	Percentage (%)
Excellent	48	80
Good	12	20
Fair	0	0
Poor	0	0

## Discussion

TKA is widely regarded as one of the most successful orthopedic procedures for the treatment of advanced knee osteoarthritis, providing substantial pain relief, deformity correction, and improvement in functional outcomes. However, optimal postoperative outcomes depend on several factors, among which precise restoration of the mechanical axis and proper component positioning are considered critical determinants of implant longevity and functional recovery [2,3]. In the present study, the majority of patients belonged to the 61-70 year age group with a slight female predominance, which is consistent with the epidemiological pattern of knee osteoarthritis reported in previous studies [3]. Hunter and Bierma‑Zeinstra reported that knee osteoarthritis is more prevalent among elderly individuals and females, largely due to hormonal, biomechanical, and lifestyle factors [1]. Comparable demographic patterns have also been reported in studies evaluating outcomes after TKA in Asian populations.

A key observation in the present study was that varus deformity was the most frequently observed preoperative alignment abnormality, present in the majority of the patients. This finding is consistent with the observations of Sharma et al., who demonstrated that medial compartment degeneration leading to progressive varus deformity represents the predominant pathological pattern in knee osteoarthritis [7]. Several studies have also shown that most patients undergoing primary TKA have varus malalignment preoperatively [10]. The use of a full-length standing orthoscanogram in the present study enabled accurate evaluation of overall lower limb alignment. Conventional knee radiographs may underestimate coronal plane deformity because they do not include the hip and ankle joints in the same image. Moreland et al. demonstrated that full-length radiographs provide a more accurate assessment of the mechanical axis and are therefore considered the gold standard for evaluating alignment after TKA [17]. The present study also highlights the value of the orthoscanogram in objectively documenting preoperative deformity and the degree of postoperative correction.

Our results demonstrated a significant increase in KSCS and KSFS at the six-month postoperative follow-up. The mean Knee Society Clinical Score improved from 41.6 ± 6.4 preoperatively to 89.3 ± 7.2 postoperatively, while the Functional Score improved from 39.2 ± 7.1 to 86.7 ± 8.0, and both improvements were statistically significant. These findings are consistent with earlier studies by Insall et al. and Bourne et al., who reported marked postoperative improvements in Knee Society scores after successful primary TKA [3,4]. A primary objective of this study was to evaluate the relationship between postoperative mechanical axis alignment and functional outcome. Our findings demonstrated that patients in whom the mechanical axis passed through Zone 2 or the central zone (Zone C) of the tibial plateau achieved significantly superior functional outcomes compared with patients with residual malalignment. More than 85% of patients with near-neutral mechanical alignment achieved excellent functional outcomes.

These findings support the well-established concept that restoration of near-neutral mechanical alignment is essential for optimal load distribution across the prosthesis [5]. Jeffery et al. demonstrated that knees aligned within ±3° of the neutral mechanical axis had significantly lower rates of implant loosening and failure compared with knees with malalignment [8]. Similarly, Ritter et al. reported that proper coronal alignment is closely associated with improved implant survivorship and superior long-term outcomes following TKA [9]. Biomechanical studies have also emphasized that varus or valgus malalignment leads to asymmetric loading of the tibial component, resulting in increased polyethylene wear and progressive periprosthetic bone loss [6,7]. Fang et al. observed that postoperative malalignment significantly increases the likelihood of early prosthetic failure due to uneven stress distribution across the prosthesis [6].

The present study supports these biomechanical concepts by demonstrating that patients with near-neutral alignment achieved better functional recovery. Several contemporary studies have also evaluated the influence of component alignment on functional outcomes after TKA [13,14]. Patil et al. reported that patients with mechanical alignment restored within ±3° of neutral had significantly higher functional scores compared with those with persistent deformity [15]. Similarly, Cooke et al. highlighted the importance of accurate restoration of the mechanical axis to ensure even load transmission and enhance implant longevity [10]. Another notable finding in the present study was the absence of major complications such as infection, periprosthetic fracture, implant loosening, or revision surgery during the follow-up period. Minor postoperative complaints, such as transient stiffness and mild pain, were observed in a few patients, but these symptoms improved with physiotherapy. Similar low complication rates have been reported in several studies evaluating outcomes after primary TKA performed with careful restoration of limb alignment [15].

Limitations

Despite these encouraging results, several limitations of the present study must be acknowledged. First, the follow-up duration was relatively short, which limits the assessment of long-term implant survival and late complications associated with malalignment. Second, the study assessed only coronal plane alignment using an orthoscanogram, whereas rotational alignment of the femoral and tibial components, which can also affect functional outcomes, was not evaluated using advanced imaging modalities such as computed tomography. Third, although the sample size was sufficient to detect short-term associations, larger multicenter studies would improve the generalizability of these findings. Additionally, surgeries were performed by different surgeons, which could introduce variability in surgical technique despite adherence to standardized operative protocols.

Future research directions

Future research should focus on studies with longer follow-up periods and larger sample sizes, the use of three-dimensional imaging techniques to assess rotational alignment, and comparisons between conventional instrumentation and computer-assisted or robotic navigation systems in achieving optimal mechanical alignment.

## Conclusions

Accurate restoration of the mechanical axis and appropriate component positioning play a crucial role in determining functional outcomes following primary TKA. The present study demonstrates that patients in whom the postoperative mechanical axis, assessed using an orthoscanogram, passed through Zone 2 and the central zone of the tibial plateau achieved significantly better clinical and functional outcomes, as measured by the Knee Society scoring system. The orthoscanogram proved to be a reliable method for evaluating lower limb alignment and component positioning. Emphasis on meticulous surgical technique aimed at achieving near-neutral mechanical alignment may improve postoperative function, patient satisfaction, and potentially enhance long-term implant survival in primary TKA.
